# Quantification of the individual risk of each Gleason pattern, including tertiary Gleason pattern 5, after radical prostatectomy: development of the modified Gleason grade grouping (mGGG) model

**DOI:** 10.1186/s12885-020-06880-8

**Published:** 2020-05-01

**Authors:** Satoru Taguchi, Yukari Uemura, Tetsuya Fujimura, Teppei Morikawa, Akihiro Naito, Taketo Kawai, Motofumi Suzuki, Haruki Kume, Hiroshi Fukuhara

**Affiliations:** 1 Department of Urology, Kyorin University School of Medicine, 6-20-2 Shinkawa, Mitaka, Tokyo, 181-8611 Japan; 2 Department of Urology, Graduate School of Medicine, The University of Tokyo, 7-3-1 Hongo, Bunkyo-ku, Tokyo, 113-8655 Japan; 3 Biostatistics Section, Department of Data Science, Center of Clinical Sciences, National Center for Global Health and Medicine, 1-21-1, Toyama, Shinjyuku-ku, Tokyo, 162-8655 Japan; 4 Biostatistics Division, Central Coordinating Unit, Clinical Research Support Center, The University of Tokyo Hospital, 7-3-1 Hongo, Bunkyo-ku, Tokyo, 113-8655 Japan; 5 Department of Urology, Jichi Medical University, Yakushiji 3311-1, Shimotsuke, Tochigi, 329-0498 Japan; 6 Department of Pathology, Graduate School of Medicine, The University of Tokyo, 7-3-1 Hongo, Bunkyo-ku, Tokyo, 113-8655 Japan

**Keywords:** Gleason grade grouping, Gleason score, Prognostic model, Prostate cancer, Prostatectomy, Quantification

## Abstract

**Background:**

While the new Gleason grade grouping (GGG), which started in 2016, has been widely validated in prostate cancer, it does not incorporate the concept of tertiary Gleason pattern 5. Furthermore, no study has “quantified” the individual risk of each Gleason pattern, including tertiary Gleason pattern 5, after radical prostatectomy.

**Methods:**

We reviewed 1022 men with adjuvant-treatment-naïve prostate cancer who underwent radical prostatectomy between 2005 and 2017. The primary endpoint was biochemical recurrence-free survival, defined as two consecutive prostate-specific antigen measurements ≥0.2 ng/ml after surgery. The individual quantitative risk score (IQRS) of each amount (primary/secondary/tertiary) of each Gleason pattern (3/4/5) was calculated using the Cox regression model. On the basis of the IQRS, the modified Gleason grade grouping (mGGG) model was developed. As a robustness analysis of the mGGG model, salvage treatment-free survival was also assessed.

**Results:**

During a median follow-up of 45 months, 229 of 1022 (22.4%) patients developed biochemical recurrence. The IQRS of each Gleason pattern was as follows: primary 5, 1.81 points (hazard ratio [HR] 6.13); secondary 5, 1.37 points (HR 3.92); tertiary 5, 0.87 points (HR 2.39); primary 4, 1.07 points (HR 2.91); secondary 4, 0.79 points (HR 2.21); and any Gleason pattern 3, 0 points (HR 1). Based on the IQRS, the mGGG model was developed, which classified patients into the following five groups: I (3 + 3 or less); II (3 + 4); III (4 + 3); IV (3 + 4 + t5, 4 + 3 + t5, 3 + 5, 5 + 3, and 4 + 4); V (4 + 4 + t5, 4 + 5, 5 + 4, and 5 + 5). The c-index for biochemical recurrence-free survival was significantly improved from 0.655 of the original GGG model to 0.672 of the mGGG model (*P* < 0.05). In the robustness analysis, the c-index for salvage treatment-free survival was also significantly improved from 0.619 of the original GGG model to 0.638 of the mGGG model (*P* < 0.05).

**Conclusions:**

The quantitative risk of tertiary (< 5%) Gleason pattern 5 is slightly higher than that of secondary (5–50%) Gleason pattern 4. Our newly developed mGGG model more accurately predicts outcomes after radical prostatectomy than the original GGG model.

## Background

Since the Gleason scoring system for pathological diagnosis of prostate cancer (PC) was published in 1966 [1], it has been widely used in clinical practice and has evolved over time. The concept of tertiary (< 5%) Gleason pattern 5 [2] has been implemented since the 2005 International Society of Urological Pathology (ISUP) Consensus Conference on Gleason Grading of Prostatic Carcinoma [3]. The prognostic significance of tertiary Gleason pattern 5 has been widely validated in various settings of PC [4–16]. In 2016, the new Gleason grade grouping (GGG) [17] started to be used in clinical practice, according to the 2014 ISUP Consensus Conference on Gleason Grading of Prostatic Carcinoma [18]. Although the prognostic value of GGG has been confirmed in several settings [19–21], it does not incorporate the concept of tertiary Gleason pattern 5. A recent study reported that integrating tertiary Gleason pattern 5 into GGG improved the accuracy of predicting the patient’s outcome after radical prostatectomy [16]. However, no study has assessed the actual effect of tertiary Gleason pattern 5 on outcomes of patients with PC. Therefore, the present study aimed to quantify the risk of each Gleason pattern, including tertiary Gleason pattern 5, after radical prostatectomy and to develop the modified Gleason grade grouping (mGGG) model.

## Methods

**Fig. 1:**
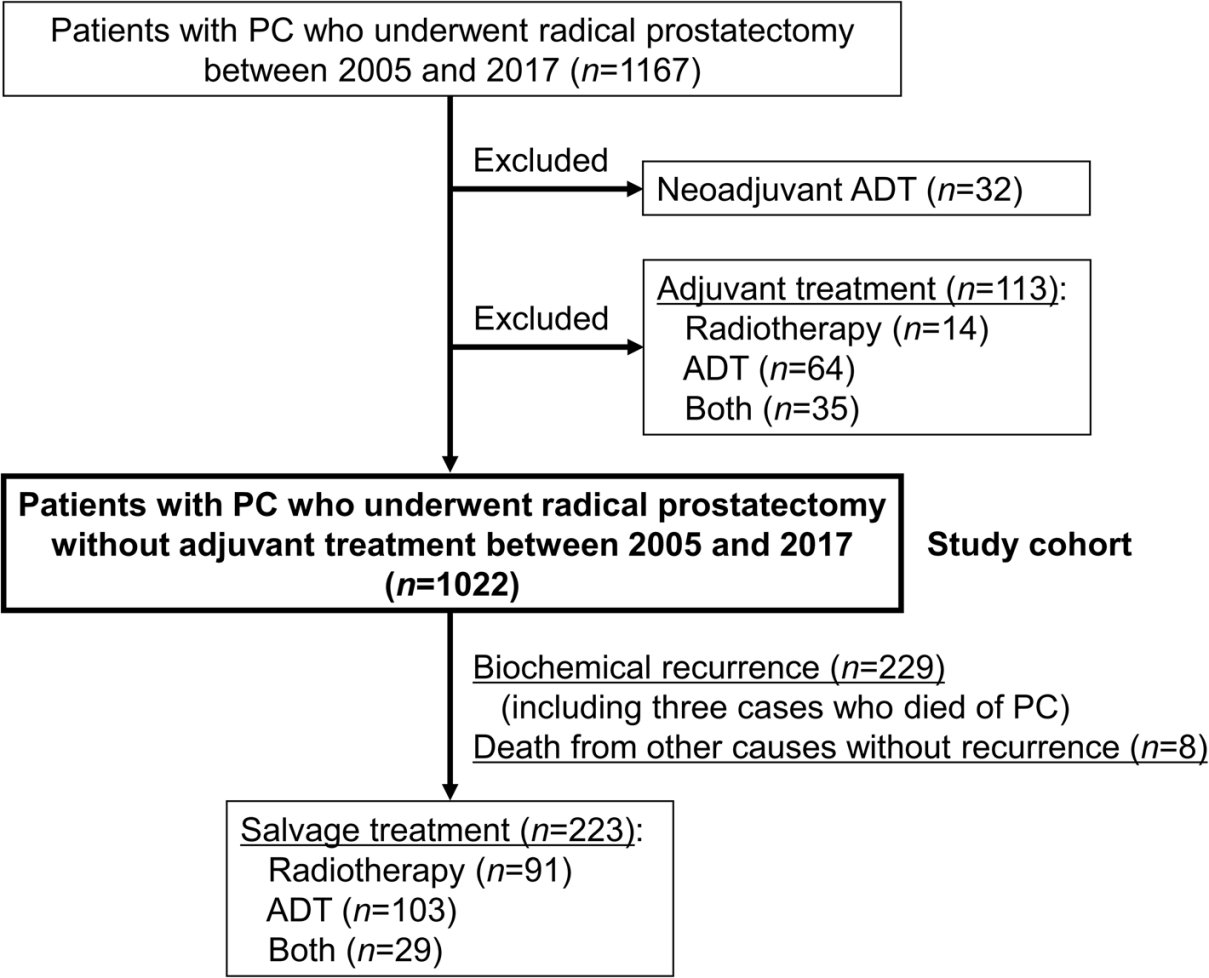
Flow chart representing the study selection process

The internal institutional review board of the Graduate School of Medicine and Faculty of Medicine, The University of Tokyo approved this retrospective study (approval number: 3124). We reviewed 1167 patients with PC who underwent radical prostatectomy at The University of Tokyo Hospital between 2005 and 2017. We excluded 32 patients who had received neoadjuvant androgen deprivation therapy (ADT) to guarantee an accurate pathological diagnosis. We also excluded 113 patients who received adjuvant treatment after surgery (radiotherapy, *n* = 14; ADT, *n* = 64; and both, *n* = 35) to assess the pure oncological outcome of surgery alone. Eventually, 1022 adjuvant-treatment-naïve patients were available for analysis (Fig. 1). All prostatectomy specimens were pathologically reviewed according to the 2005 ISUP Consensus Conference on Gleason Grading of Prostatic Carcinoma [3].

The primary endpoint was biochemical recurrence-free survival (BRFS), defined as two consecutive prostate-specific antigen (PSA) measurements ≥0.2 ng/ml after surgery [22]. Salvage radiotherapy and/or ADT was implemented when biochemical and/or radiological recurrence was observed [22–25]. We used Kaplan–Meier analysis with the log-rank test to determine survival differences among categories. Univariate and multivariate Cox proportional hazards regression models were also used to predict outcomes. Follow-up information was obtained as of March 2018.

The individual quantitative risk score (IQRS, points) of each amount (primary/secondary/tertiary) of each Gleason pattern (3/4/5) was calculated using the Cox regression model, and reported with the corresponding hazard ratio (HR). The HR was calculated from the IQRS by the following formula: HR = *e*^IQRS^. The IQRS of any Gleason pattern 3 was set as 0 points (HR 1), because a Gleason score (GS) of 3 + 3 rarely correlates with PC-specific death and metastasis, despite being pathologically malignant [26, 27]. On the basis of the IQRS, the mGGG model was finally developed. Concordance (c) indices were calculated both for the mGGG model and the original GGG model. As a robustness analysis of the newly developed mGGG model, we also assessed another endpoint of salvage treatment-free survival.

All statistical analyses were performed using JMP Pro version 14.0.0 and SAS version 9.4 (SAS Institute, Cary, NC, USA). *P* < 0.05 was considered to indicate a significant difference.

## Results

Patients’ characteristics are shown in Table 1. Findings of extraprostatic extension and seminal vesicle invasion were included in pathological T stage as pT3a and pT3b, respectively. During a median follow-up of 45 (interquartile range: 21–77) months, 229 of 1022 (22.4%) patients developed biochemical recurrence, and of them, three died of PC. Eight of 1022 (0.8%) patients died from other causes without recurrence, resulting in 237 events for BRFS (Fig. 1).

**Table 1:** Patient characteristics (*n* = 1022)

Parameter	Value
Age at surgery, years, median (interquartile range)	67 (62–71)
Surgical technique, no. (%):	
Open	414 (40.5)
Laparoscopic	32 (3.1)
Robotic	576 (56.4)
Initial PSA, ng/ml, median (interquartile range)	7.7 (5.6–11.2)
Pathological GS, no. (%):	
3 + 3 or less	119 (11.6)
3 + 4	395 (38.7)
3 + 4 + t5	53 (5.2)
3 + 5	17 (1.7)
4 + 3	148 (14.5)
4 + 3 + t5	94 (9.2)
4 + 4	59 (5.8)
4 + 4 + t5	8 (0.8)
4 + 5	108 (10.6)
5 + 3	2 (0.2)
5 + 4	19 (1.9)
5 + 5	0 (0)
Pathological T stage, no. (%):	
pT2	708 (69.3)
pT3a	270 (26.4)
pT3b	42 (4.1)
pT4	2 (0.2)
Pathological N stage, no. (%):	
pN0/x	1012 (99.0)
pN1	10 (1.0)
Resection margin, no. (%)	355 (34.7)
Median follow-up, months (interquartile range)	45 (21–77)

*PSA* Prostate-specific antigen, *GS* Gleason score

**Fig. 2:**
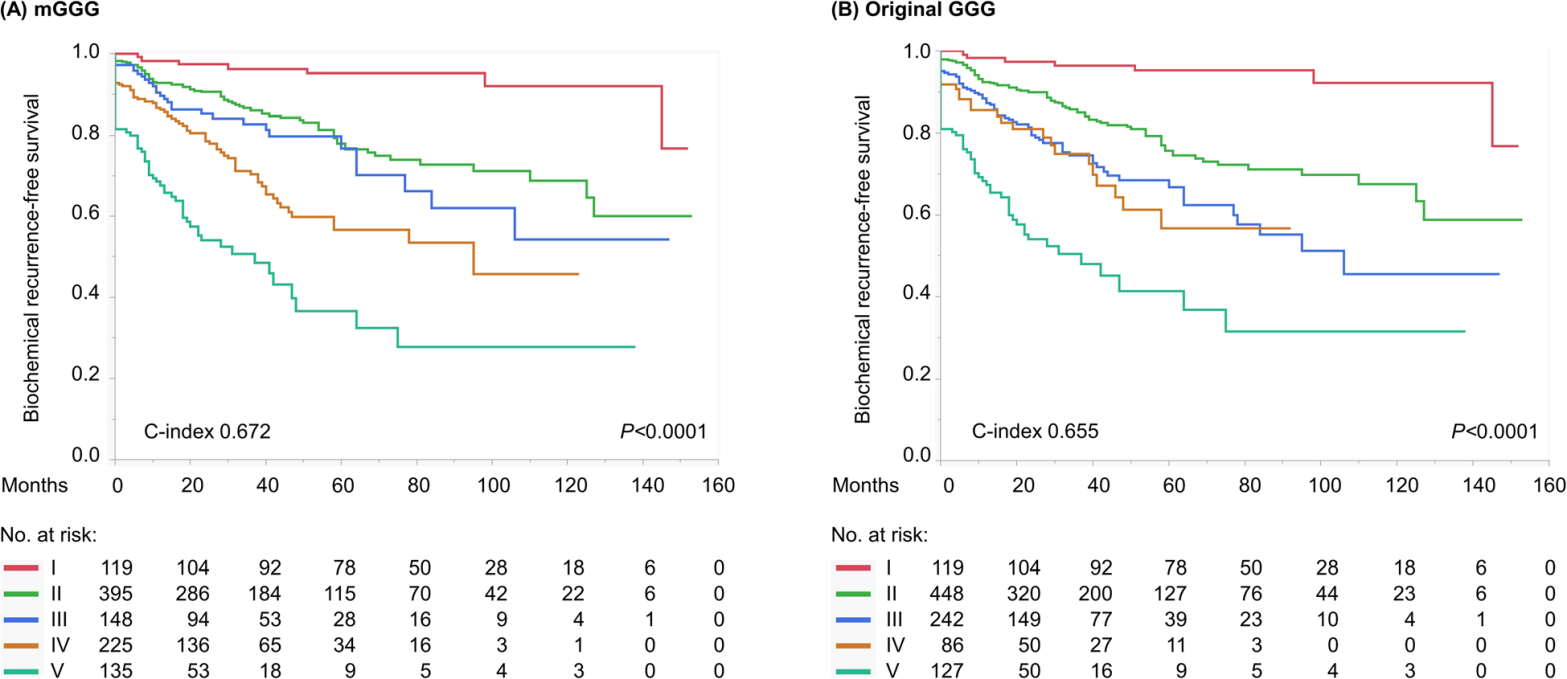
Kaplan–Meier curves depicting biochemical recurrence-free survival (BRFS) according to (**a**) the modified Gleason grade grouping (mGGG) model and (**b**) the original Gleason grade grouping (GGG) model (log-rank test, both *P* < 0.0001)

The IQRS of each Gleason pattern was as follows: primary 5, 1.81 points (HR 6.13); secondary 5, 1.37 points (HR 3.92); tertiary 5, 0.87 points (HR 2.39); primary 4, 1.07 points (HR 2.91); secondary 4, 0.79 points (HR 2.21); and any Gleason pattern 3, 0 points (HR 1) (Table 2). On the basis of the IQRS, we developed the mGGG model, which classified patients into five groups (Table 3): I (3 + 3 or less); II (3 + 4); III (4 + 3); IV (3 + 4 + t5, 4 + 3 + t5, 3 + 5, 5 + 3, and 4 + 4); V (4 + 4 + t5, 4 + 5, 5 + 4, and 5 + 5). There were significant differences in the survival profiles of the five groups of the mGGG model (*P* < 0.0001) (Fig. 2a), whereas the discrimination between group III and group IV of the original GGG model seemed insufficient (Fig. 2b). The mGGG model achieved a significantly higher predictive accuracy for BRFS (c-index: 0.672) than the original GGG model (c-index: 0.655) (*P* = 0.047). Univariate and multivariate Cox proportional hazards regression analyses for BRFS are shown in Table 4. Even after taking into account clinicopathological confounding factors in multivariate analysis, the mGGG model was predictive and prognostic in a score-dependent manner.

**Table 2:** The individual quantitative risk score (IQRS, points) and hazard ratio (HR) of each Gleason pattern (3/4/5) and its echelon (primary/secondary/tertiary)

	Pattern 5	Pattern 4	Pattern 3 or less
Primary	1.81 points (HR 6.13)	1.07 points (HR 2.91)	0 points (HR 1)
Secondary	1.37 points (HR 3.92)	0.79 points (HR 2.21)	0 points (HR 1)
Tertiary	0.87 points (HR 2.39)		

HR is calculated from the IQRS by the following formula: HR = *e*^IQRS^

**Table 3:** The modified Gleason grade grouping (mGGG) model based on the individual quantitative risk score (IQRS, points) of each pathological Gleason score (GS)

mGGG	Pathological GS (original GGG category)	*n*	IQRS, points	HR
**I**	3 + 3 or less (I)	119	0	1
**II**	3 + 4 (II)	395	0.79	2.21
**III**	4 + 3 (III)	148	1.07	2.91
**IV**	3 + 4 + t5 (II)	53	1.67	5.29
	4 + 3 + t5 (III)	94	1.94	6.96
	3 + 5 (IV)	17	1.37	3.92
	5 + 3 (IV)	2	1.81	6.13
	4 + 4 (IV)	59	1.86	6.45
**V**	4 + 4 + t5 (IV)	8	2.73	15.40
	4 + 5 (V)	108	2.44	11.43
	5 + 4 (V)	19	2.61	13.57
	5 + 5 (V)	0	3.18	24.06

*mGGG* modified Gleason grade grouping, *GS* Gleason score, *GGG* Gleason grade grouping *IQRS* Individual quantitative risk score, *HR* Hazard ratio

**Table 4:** Univariate and multivariate Cox proportional hazards regression analyses for biochemical recurrence-free survival (BRFS)

Variable	Univariate regression		Multivariate regression	
	HR (95% CI)	*P*	HR (95% CI)	*P*
Age at surgery (continuous)	1.00 (0.98 to 1.02)	0.98	0.97 (0.95 to 0.99)	0.02*
Surgical technique:		< 0.01*		< 0.01*
Open	Reference	–	Reference	–
Laparoscopic	0.49 (0.19 to 1.02)	0.06	0.72 (0.28 to 1.52)	0.42
Robotic	0.64 (0.48 to 0.84)	< 0.01*	0.59 (0.43 to 0.81)	< 0.01*
Initial PSA:		< 0.01*		0.22
< 10 ng/ml	Reference	–	Reference	–
10–20 ng/ml	1.54 (1.15 to 2.03)	< 0.01*	1.25 (0.93 to 1.67)	0.13
≥ 20 ng/ml	2.76 (1.81 to 4.07)	< 0.01*	1.30 (0.84 to 1.94)	0.23
mGGG model:		< 0.01*		< 0.01*
I	Reference	–	Reference	–
II	4.53 (2.06 to 9.92)	< 0.01*	4.54 (2.07 to 9.96)	< 0.01*
III	5.93 (2.58 to 13.63)	< 0.01*	5.79 (2.50 to 13.44)	< 0.01*
IV	10.22 (4.64 to 22.52)	< 0.01*	9.37 (4.20 to 20.86)	< 0.01*
V	21.28 (9.61 to 47.09)	< 0.01*	19.26 (8.50 to 43.66)	< 0.01*
Pathological T stage:		< 0.01*		< 0.01*
pT2	Reference	–	Reference	–
pT3a	2.85 (2.18 to 3.72)	< 0.01*	1.56 (1.17 to 2.09)	< 0.01*
pT3b	5.34 (3.12 to 8.60)	< 0.01*	2.25 (1.25 to 3.85)	< 0.01*
pT4	2.59 (0.15 to 11.75)	0.41	3.20 (0.18 to 15.60)	0.34
Pathological N stage:		< 0.01*		< 0.01*
pN0/x	Reference	–		–
pN1	11.88 (5.59 to 22.13)	< 0.01*	5.92 (2.71 to 11.49)	< 0.01*
Resection margin	3.48 (2.68 to 4.56)	< 0.01*	2.25 (1.67 to 3.04)	< 0.01*

*HR* Hazard ratio, *CI* Confidence interval, *PSA* Prostate-specific antigen, *mGGG* modified Gleason grade grouping

*Statistically significant

**Fig. 3:**
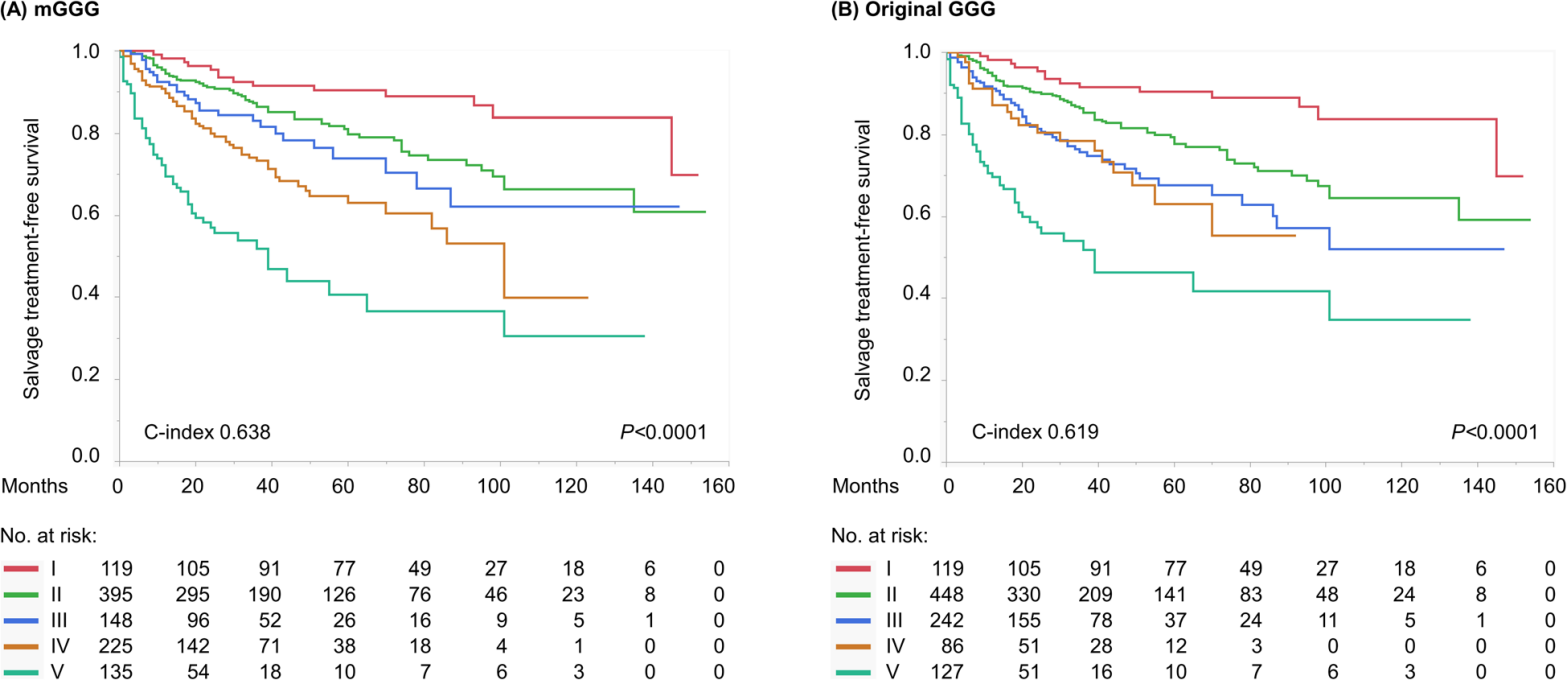
Kaplan–Meier curves depicting salvage treatment-free survival (as a robustness analysis) according to (**a**) the modified Gleason grade grouping (mGGG) model and (**b**) the original Gleason grade grouping (GGG) model (log-rank test, both *P* < 0.0001)

In the robustness analysis assessing salvage treatment-free survival, significant differences were observed in the survival profiles of the five groups of the mGGG model (*P* < 0.0001) (Fig. 3a), whereas the discrimination between group III and group IV of the original GGG model seemed insufficient (Fig. 3b). Also for salvage treatment-free survival, the mGGG model achieved a significantly higher predictive accuracy (c-index: 0.638) than the original GGG model (c-index: 0.619) (*P* = 0.029).

## Discussion

To the best of our knowledge, the present study is the first quantification of the individual risk of each Gleason pattern, including tertiary Gleason pattern 5, after radical prostatectomy. Notably, the quantitative risk of tertiary (< 5%) Gleason pattern 5 was slightly higher than that of secondary (5–50%) Gleason pattern 4. Furthermore, our newly developed mGGG model more accurately predicted outcomes after radical prostatectomy than the original GGG model.

The pathological diagnostic system of PC is unique and complex in that it considers the worst and second worst lesions, as well as their percentages of areas [1, 3, 18], whereas those of other cancers usually simply use the worst lesion. As described above, the Gleason scoring system, which was published in 1966 [1], has evolved over time. The concept of tertiary (< 5%) Gleason pattern 5 [2] has been implemented since 2005 [3] and the GGG [17] has been proposed since 2014 [18], although it does not incorporate the concept of tertiary Gleason pattern 5. Recently, a Japanese research group reported that integrating tertiary Gleason pattern 5 into GGG improved the prediction accuracy of biochemical recurrence after radical prostatectomy using a retrospective cohort of 1000 patients [16]. Similarly, Lucca et al. previously investigated the significance of tertiary Gleason pattern 5 in a large cohort (*n* = 4146) of patients with localized GS 7 PC treated by radical prostatectomy [13]. They identified the presence of tertiary Gleason pattern 5 as an independent predictor of biochemical recurrence and developed a prognostic model for 416 patients with GS 7 (3 + 4 or 4 + 3) and tertiary Gleason pattern 5. This study judiciously highlighted the importance of tertiary Gleason pattern 5 among patients with GS 7, however, it did not evaluate the relative importance of tertiary Gleason pattern 5 in patients with all GS categories. Actually, no study so far has “quantified” the actual effect of tertiary Gleason pattern 5 on outcomes after radical prostatectomy. In this context, the present study clearly identified the IQRS of each Gleason pattern (3/4/5) and its echelon (primary/secondary/tertiary) (Table 2). Notably, the IQRS of tertiary (< 5%) Gleason pattern 5 was slightly higher than that of secondary (5–50%) Gleason pattern 4. This finding is in accordance with previous reports, which emphasized the importance of (tertiary) Gleason pattern 5 in various settings of PC [4–16]. The present study confirmed the paramount importance of Gleason pattern 5, regardless of its amount, in predicting outcomes after surgery.

On the basis of the IQRS of each Gleason pattern, the present study developed the mGGG model, in which the original GGG model was used as a backbone with GS 3 + 4 + t5 (originally group II) and GS 4 + 3 + t5 (originally group II) shifted into group IV and with GS 4 + 4 + t5 (originally group IV) shifted into group V (Table 3). Given that the mGGG model enables accurate prediction of BRFS after surgery with a higher c-index than the original GGG model, we deem that the mGGG model is a better scoring system than the original GGG. Additionally, the robustness of the mGGG model was confirmed by assessing salvage treatment-free survival, a treatment-oriented endpoint, other than BRFS, a pure oncological endpoint. Recently, the importance of treatment-oriented endpoints has been recognized in the field of oncology and some endpoints such as ADT-free survival have already been assessed in studies of PC [28]. Both for BRFS and salvage treatment-free survival, the predictive accuracy of the mGGG model was higher than that of the original GGG model. This might mean the usefulness of the mGGG model in the real-world clinical practice.

This study had several limitations. Firstly, it was a retrospective analysis of a single institution with a relatively short follow-up. Secondly, the pathological review of prostatectomy specimens was based on the 2005, but not 2014, ISUP Consensus Conference on Gleason Grading of Prostatic Carcinoma, although their criteria were generally identical except for some minor revisions. Thirdly, we could not assess GS 3 + 3 with tertiary Gleason pattern 5 and GS 5 + 5 because of lack of applicable cases. Given that the IQRS for GS 3 + 3 + t5 and GS 5 + 5 are theoretically 0.87 points (HR 2.39) and 3.18 points (HR 24.06), respectively, GS 3 + 3 + t5 and GS 5 + 5 would be assigned to the mGGG category II and V, respectively. Further studies should be conducted to externally validate the mGGG model and to finally establish the optimal pathological diagnostic system of PC.

## Conclusions

The quantitative risk of tertiary (< 5%) Gleason pattern 5 is slightly higher than that of secondary (5–50%) Gleason pattern 4. Our newly developed mGGG model more accurately predicts outcomes after radical prostatectomy than the original GGG model.

## Data Availability

Due to ethical restrictions, the raw data underlying this study are available from the corresponding author upon reasonable request.

## Abbreviations

ADT: Androgen deprivation therapy
GGG: Gleason grade grouping
GS: Gleason score
HR: Hazard ratio
IQRS: Individual quantitative risk score
ISUP: International Society of Urological Pathology
mGGG: Modified Gleason grade grouping
PC: Prostate cancer
PSA: Prostate-specific antigen
